# Association of Subjective Cognitive Concerns With Performance on Mobile App–Based Cognitive Assessment in Cognitively Normal Older Adults: Observational Study

**DOI:** 10.2196/64033

**Published:** 2025-02-04

**Authors:** Caroline O Nester, Alyssa N De Vito, Sarah Prieto, Zachary J Kunicki, Jennifer Strenger, Karra D Harrington, Nelson Roque, Martin J Sliwinski, Laura A Rabin, Louisa I Thompson

**Affiliations:** 1 Department of Psychiatry and Human Behavior, The Warren Alpert Medical School of Brown University, 222 Richmond St, Providence, RI, 02903, United States, 1 (401) 863-3330; 2 Memory and Aging Program, Butler Hospital, Providence, RI, United States; 3 Center for Healthy Aging, Penn State University, University Park, PA, United States; 4 Department of Psychology, University of Central Florida, Orlando, FL, United States; 5 Department of Psychology, Brooklyn College CUNY, Brooklyn, NY, United States

**Keywords:** subjective cognitive concerns, subjective cognitive decline, digital cognitive assessment, mobile app, app-based, preclinical Alzheimer disease, mild cognitive impairment, MCI, preclinical dementia, mobile monitoring of cognitive change, Cognitive Function Instrument, mHealth, mobile health, applications, cognition, assessment, remote, geriatrics, gerontology, aging, memory, older adult, elderly, digital health, mobile phone

## Abstract

**Background:**

Subjective cognitive concerns (SCCs) may be among the earliest clinical symptoms of dementia. There is growing interest in applying a mobile app–based cognitive assessment to remotely screen for cognitive status in preclinical dementia, but the relationship between SCC and relevant mobile assessment metrics is uncertain.

**Objective:**

This study aimed to characterize the relationship between SCC and adherence, satisfaction, and performance on mobile app assessments in cognitively unimpaired older adults.

**Methods:**

Participants (N=122; Mean_age_=68.85 [SD 4.93] years; Mean_education_=16.85 [SD 2.39] years; female: n=82, 66.7%; White:n=106, 86.2%) completed 8 assessment days using Mobile Monitoring of Cognitive Change (M2C2), an app-based testing platform, with brief daily sessions within morning, afternoon, and evening time windows (24 total testing sessions). M2C2 includes digital working memory, processing speed, and episodic memory tasks. Participants provided feedback about their satisfaction and motivation related to M2C2 upon study completion. SCC was assessed using the Cognitive Function Instrument. Regression analyses evaluated the association between SCC and adherence, satisfaction, and performance on M2C2, controlling for age, sex, depression, and loneliness. Linear-mixed effects models evaluated whether SCC predicted M2C2 subtest performance over the 8-day testing period, controlling for covariates.

**Results:**

SCC was not associated with app satisfaction or protocol motivation, but it was significantly associated with lower rates of protocol adherence (ß=−.20, *P*=.37, 95% CI −.65 to −.02). Higher SCC endorsement significantly predicted worse overall episodic memory performance (ß=−.20, *P*=.02, 95% CI −.02 to −.01), but not working memory or processing speed. There was a main effect of SCC on working memory performance at day 1 (estimate=−1.05, SE=0.47, *P*=.03) and a significant interaction between SCC and working memory over the 8-day period (estimate=0.05, SE=0.02, *P*=.03), such that SCC was associated with initially worse, then progressively better working memory performance.

**Conclusions:**

SCCs are associated with worse overall memory performance on mobile app assessments, patterns of cognitive inefficiency (variable working memory), and mildly diminished adherence across an 8-day assessment period. Findings suggest that mobile app assessments may be sensitive to subtle cognitive changes, with important implications for early detection and treatment for individuals at risk for dementia.

## Introduction

Subjective cognitive decline (SCD) refers to self-perceived changes in cognitive functioning in the setting of normative performance on objective cognitive measures [1]. Older adults with SCD are more likely to demonstrate cognitive decline over time compared with their peers who do not endorse significant cognitive concerns [1-6]. This association has led many to posit that SCD may be one of the earliest clinical manifestations of Alzheimer disease and related dementias (ADRD) [1,3]. SCD has been linked with critical ADRD neural substrates and biomarkers, including structural [7-9] and functional [4,10] alterations, white matter dysfunction [11-13], and the presence of amyloid (Aβ) and tau [14-17]. However, it is important to acknowledge that most individuals notice changes in cognition as they age (up to 80% of people over the age of 70 years), and that many individuals with SCD do not ultimately progress to dementia [2]. As such, investigating novel markers within SCD may enhance early risk detection in this preclinical population.

Disentangling the relationship between SCD and performance on traditional neuropsychological measures is inherently complex. By definition, individuals with SCD perform in the normal range on objective cognitive tasks [3]. However, some studies have demonstrated that SCD is not without mild cognitive deficits. For example, there is evidence of mild reductions in processing speed, executive functioning, language, and memory in SCD [18-22], and such minor deficits have been correlated with concurrent Alzheimer disease (AD) biomarkers and future clinical progression [23]. There is presently a multitude of approaches in the field and widespread debate about how to best assess SCD, and measure selection may directly impact the association of SCD with concurrent cognition and risk for future cognitive decline [24-27]. It is also essential to recognize that SCD intrinsically reflects a longitudinal change over time [28]; however, traditional neuropsychological evaluations only capture a snapshot of in-the-moment cognitive status. Furthemore, for individuals with a high cognitive baseline, normative performance on objective testing may actually represent a decline in cognitive performance [1]. Novel and highly sensitive cognitive tools are needed to characterize more subtle deficits experienced by individuals with SCD [28].

There is growing interest in using smartphone-based, digital technology to remotely screen and track cognitive functioning in older adults at risk for dementia or those along the AD continuum. Smartphone usage among older Americans is highly prevalent (62%‐81% of people over the age of 60 use a smartphone) [29], and a number of smartphone-based cognitive assessment apps have been developed [30,31]. Such approaches are not only more accessible, convenient, time-effective, and scalable [31-33], but also may be more sensitive to subtle cognitive changes not captured by traditional paper and pencil cognitive assessments [31,32]. Smartphone-based cognitive assessments may also demonstrate superior ecological validity when completed in the individual’s lived context with a familiar piece of technology [30,31,34]. Smartphone technology also facilitates high-frequency, repeated cognitive assessment, which allows for the quantification of subtle markers of decline in older adults, such as intraindividual variability and patterns in cognition over time [30-32,35,36]. However, the self-administered, unsupervised nature of smartphone-based cognitive assessments raises concerns related to task adherence, engagement, and potentially confounding factors that may occur within an uncontrolled environment [32].

Particularly relevant to aging research is the limited understanding of how subjective cognitive concerns (SCCs), the core feature of SCD, may be associated with engagement, satisfaction, and performance on such smartphone-based cognitive assessments. Preliminary evidence suggests that smartphone use may be associated with fewer SCCs in older adulthood and possibly constitute a protective factor against cognitive decline [37]; hence, smartphones may represent a promising tool for novel assessments and potential interventions for at-risk populations with self-perceived cognitive decline. Emerging literature suggests that smartphone-based cognitive testing generally has adequate adherence rates (>70%) [32], but little is understood about factors (such as SCC) which may impact engagement or motivation on unsupervised digital tests [38]. These are especially important relationships to disentangle, given the ubiquity of SCC in aging populations [2], high rates of smartphone usage [29], and growing interest in remote assessment to enhance accessibility to cognitive screening. In addition, the relationships between digital cognitive testing and AD biomarkers [39-41], as well as SCC and AD biomarkers [15,17,42], is supported by emerging research, but no studies to date have directly investigated the relationship between SCC, AD biomarkers, and digital smartphone cognitive tasks.

In light of the need to better understand the role of SCC in smartphone-based cognitive assessments in aging populations at risk for AD, the present analysis sought to characterize the relationship between SCC and adherence (aim 1), satisfaction and motivation (aim 2), and performance (aim 3) on a mobile app–based digital cognitive assessment in a sample of cognitively unimpaired older adults. Given the established association between SCC, cerebral Aβ positivity (eg, the hallmark biomarker of AD), and risk for cognitive decline [14-16,23], a secondary aim sought to understand if Aβ positivity moderated the relationship between SCC and performance on the mobile app–based cognitive assessment. Results represent the first step toward quantifying the impact of SCC on smartphone-based cognitive testing in older adult populations, with crucial implications for early detection in those at risk for cognitive decline and dementia.

## Methods

### Participants, Recruitment, and Procedures

**Figure 1.**
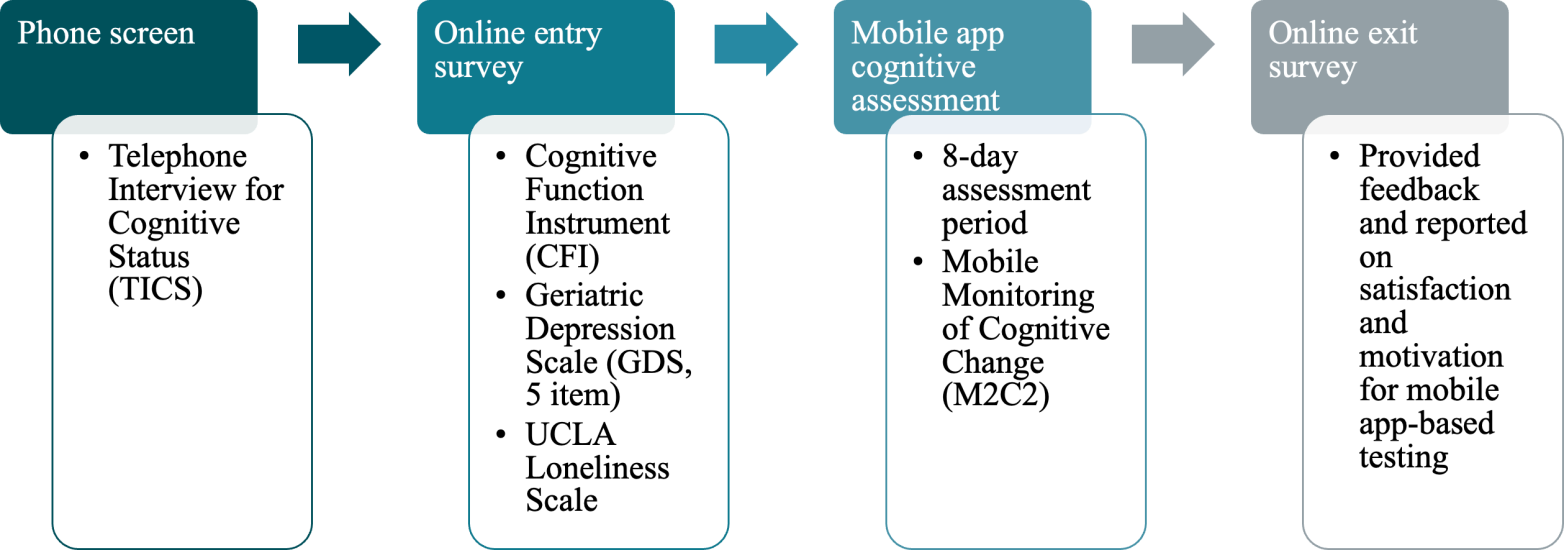
Study procedures. UCLA: University of California, Los Angeles.

Participants consisted of 122 cognitively unimpaired older adults, between the ages of 60 and 80 years of age, recruited from the Butler Alzheimer’s Prevention Registry, a local database of older adults interested in AD research at the Butler Hospital Memory and Aging Program (MAP) [43]. All study procedures were carried out remotely, and detailed recruitment and procedural information has been described previously [38,41,44]. Study procedures are presented in Figure 1.

We used a targeted recruitment to enroll individuals with previous amyloid positron emission tomography (PET) data (elevated [Aß+] or nonelevated [Aß-] determined by clinical read by a radiologist) (final n=73) [41] as well as those without PET data. PET results were available if recorded in our Registry as part of previous research participation at the MAP. Individuals (n=256) were invited to participate in the study through an email or phone call. Individuals who consented (n=146) completed an online survey before the initiation of the remote testing protocol, which included various screening measures, including the Cognitive Functioning Index (CFI) [45] (subjective cognitive concern screen), Geriatric Depression Scale 5-item version (GDS-5) [46] (depressive symptoms screen; score range 0‐5, with higher scores indicating worse depressive symptoms), and the University of California, Los Angeles (UCLA) 3-Item Loneliness Scale [47] (loneliness screen; score range 0‐6, with higher scores indicating worse loneliness). The modified Telephone Interview for Cognitive Status (TICSm) [48] (objective cognition screen; score range 0‐50, with lower scores indicating worse cognitive functioning) was also completed before the remote testing protocol by phone, administered by a research assistant. During screening, 23 participants were excluded, and 1 participant was lost to follow up. After the completion of the 8 day remote study protocol, participants (final n=122) were invited to provide feedback (including study satisfaction and motivation) on their experience through an online survey, and a US $20 gift card was provided for compensation.

Inclusion criteria involved unimpaired cognition, daily use of a smartphone, and familiarity with smartphone features. Unimpaired cognition was defined as a TICSm cutoff score of ≥34 [48]. Exclusion criteria included self-reported history of cognitive impairment or dementia, history of neurologic disease or severe mental illness, and physical inability to complete smartphone-based testing.

### Subjective Cognitive Concerns

The CFI is a 14-item self-report measure which probes changes in cognitive and functional domains over the past year. The CFI was selected for inclusion in this study as it is a widely used, briefly administered measure of SCC that has been well-validated for used in cognitively healthy and preclinical dementia populations [42,45,49,50]. Items reflect memory concerns, increased use of compensatory strategies, changes in driving, or difficulty managing instrumental activities of daily living. Participants respond with yes (1 point), no (0 points), or maybe (0.5 points), with total scores ranging from 0 to 14 (continuous variable). Higher scores reflect greater SCC. As a sensitivity analysis, we also created a SCC variable that included CFI items that solely assessed cognition (CFI-Cog), by removing the functioning related items to create a refined examination of SCC. CFI-Cog included CFI items 1, 2, 3, 4, 5, 6, 8, 11, and 13. This approach is consistent with a recent study which identified that cognitively focused CFI items, rather than functionally related CFI items, were endorsed at a higher rate and more likely to be associated with Aß+ in cognitively unimpaired older adults [42].

### Mobile App–Based Cognitive Assessment

#### Overview

**Figure 2.**
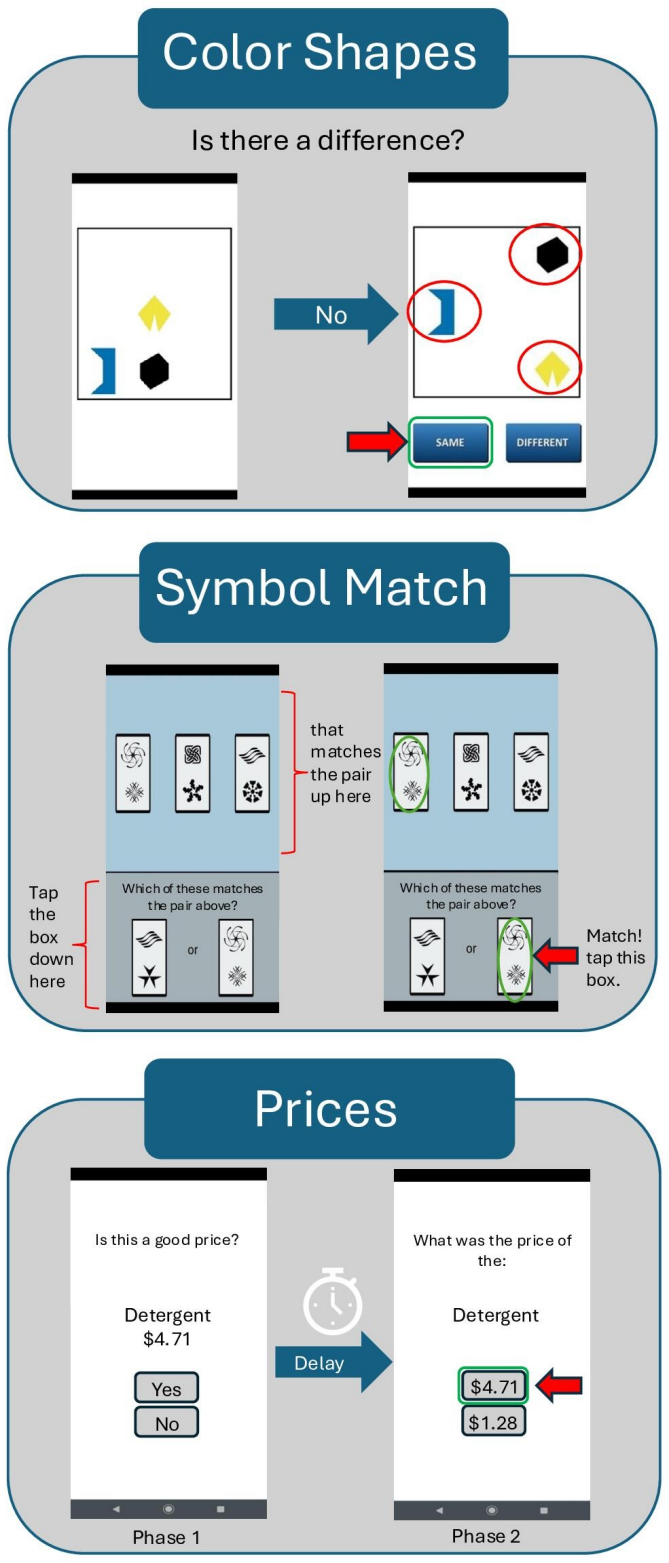
Mobile Monitoring of Cognitive Change (M2C2) app subtests.

Remote cognitive tasks were completed using the Mobile Monitoring of Cognitive Change (M2C2) app [51] (more details in Figure 2), a cognitive testing platform developed as part of the National Institute of Aging Mobile Toolbox initiative, with strong previous theoretical, empirical, and psychometric support, including evidence of sensitivity to age and age-related neuropathology [34,38,44]. Android smartphones preloaded with the cognitive assessment app were mailed to participants along with a detailed use guide. Phone functions were locked to prevent the use of features such as web browsing and the camera. Participants completed 3, brief (ie, 3‐4 minutes) M2C2 sessions each day during morning, afternoon, and evening time windows for 8 consecutive days. Additional sessions could be completed on day 9 as optional or make-up sessions. Staff provided support through phone or email as needed, as described previously [41]. During each M2C2 session, participants completed 3 previously characterized cognitive measures assessing visual working memory (Color Shapes), processing speed (Symbol Match), and episodic memory (Prices). Each task took approximately 60 seconds to complete.

#### Color Shapes

The Color Shapes visual working memory task is a visual array change detection test measuring intraitem feature binding. Participants determine if shapes change color across 2 sequential presentations in which the shape locations change. Performance was measured by discriminability (d-prime) performance calculated from the proportion of correct identifications and proportion of misidentified stimuli [51].

#### Symbol Match

The Symbol Match processing speed task is a speeded continuous performance test of conjunctive feature search. Participants are asked to identify matching symbol pairs. Performance was measured by the median reaction time to complete the task across all trials (in milliseconds) [51].

#### Prices

The Prices episodic memory task uses an immediate delayed forced-choice recognition paradigm. Participants incidentally encode 10 grocery item-price pairs for later recall while judging whether or not the item’s price is “good.” Recall trials begin immediately after the learning trials. Performance was measured by the proportion of correct responses on the 10 recall trials [51].

### Study Adherence, Satisfaction, and Motivation

Adherence was quantified as the number of testing sessions completed across the 8-day ambulatory protocol (out of 24 sessions).

Participants’ feedback covered various aspects, including their satisfaction with the protocols and their level of motivation to engage with the study tasks. Feedback related to satisfaction was measured with 2 items: “Completing the brain games was fun” and “I became bored with the brain games.” Participants rated their responses on a 6-point Likert scale (1=strongly disagree to 6=strongly agree). For the purposes of this study, responses were dichotomized by those who agreed (Likert scale responses 4, 5, and 6) or those who disagreed (Likert scale responses 1, 2, and 3) on these items due to a largely positive response bias (most individuals found the tasks to be fun and not boring).

Feedback regarding motivation was measured on the item: “It was hard to motivate myself to complete the games each time.” Participants rated their response on a 5-point Likert scale (1=Agree to 5=Disagree). As above, responses were dichotomized into those who agreed or were neutral (Likert scale responses 1, 2, and 3) and those who disagreed (Likert scale responses 4 and 5) for the purpose of this analysis due to a largely positive response bias (most individuals found themselves to be easily motivated to complete the tasks).

### Statistical Analysis

Descriptive statistics were conducted to characterize the sample in terms of demographics (self-reported biological sex, race, ethnicity, and years of education), self-reported symptoms of depression (by the Geriatric Depression Scale 5-item version [46]), loneliness (by the UCLA Loneliness Scale [47]), and SCC (CFI total and CFI-Cog), relevant app-based metrics (ie, adherence, satisfaction and motivation), and objective cognitive performance (ie, visual working memory, processing speed, and episodic memory). Pearson correlation coefficient (for continuous variables) and point biserial correlation (for binary variables) were implemented as appropriate to assess the relationship between SCC and demographic variables, depression, loneliness, and outcomes of interest (eg, adherence, satisfaction, motivation, and performance on app-based cognitive assessment). To address aims 1‐3 and secondary analyses, SCC (continuous variable) was the main predictor of interest and we adjusted for covariates, including age, sex, depression, and loneliness, in all models. Covariates were selected based on significant association with independent and dependent variables of interest. A priori significance threshold (α) was set at *P*≤.05. For our moderation analyses, we examined α levels up to *P*≤.10.

To test aim 1, linear regression models were used to evaluate the association between SCC and adherence (continuous variable) over the 8-day protocol. For the purposes of this analysis, the CFI total score was the main SCC predictor of interest. We present results for CFI-Cog only when they differ from the CFI total score. To test aim 2, logistic regression models were conducted to evaluate the association between SCC and satisfaction (dichotomized self-report items related to level of fun and boredom), and SCC and motivation (dichotomized self-report items related to motivation). Individual regression models were implemented for each motivation and satisfaction outcome of interest. To test aim 3, separate linear regression models were constructed to evaluate the association between SCC and performance on each M2C2 based cognitive assessment (Prices, Color Shapes, and Symbol Search, all continuous variables). Linear-mixed effects models were used to evaluate whether SCC predicted M2C2 subtest performance over time while controlling for age, sex, and loneliness. Finally, as a secondary analysis in a subsample of participants with Aβ PET data, we fit linear regression models with SCC and the interaction of SCC and Aβ PET positivity as predictors, to test the moderation effect of Aβ PET positivity on the association between SCC and study adherence and performance on M2C2 tasks, adjusting for covariates. All descriptive and regression analyses were conducted using SPSS version 28.0.1.1 (IBM Corp). Linear mixed-effects models were conducted using the *nlme* R package.

### Ethical Considerations

The project received approval from the Butler Hospital Institutional Review Board (#1882523), and all the participants provided informed consent. All data was de-identified. Participants were compensated for their time with a $20 gift card.

## Results

### Participant Characteristics and Correlates of Subjective Cognitive Concerns

Overall sample demographics and SCC, adherence, satisfaction, motivation, and performance variables are presented in Table 1. The sample (N=122) was 68.85 (SD 4.93) years old (range=60‐81 y) with 16.52 (SD 2.39) years of formal education. The majority of the sample identified as female (n=82, 66.7%), White (n=106, 86.2%), and non-Hispanic (n=109, 88.6%). In the subset of individuals with Aβ PET scans, there were 25 Aβ positive participants and 48 Aβ negative participants. Subjective cognitive concerns, as measured on the CFI, were fairly minimal in this sample. On average, the sample scored 1.8 (SD 1.7) out of 14 possible points on the overall CFI ; however, the most common score on the CFI was 0 (mode=0), with 18% of the sample expressing no concerns about their cognition on the CFI.

**Table 1.** Sample demographic, subjective cognitive concerns, and mobile app protocol metrics.

	Total sample (N=122)		Subsample with Aβ^a^ PET^b^ (n=73)	
**Demographic characteristics**				
Age (years), mean (SD)	68.85 (4.93)		69.25 (4.48)	
Education (years), mean (SD)	16.52 (2.39)		16.45 (2.60)	
Female, n (%)	82 (66.7)		52 (71.2)	
White, n (%)	106 (86.2)		65 (89)	
Non-Hispanic, n (%)	109 (88.6)		64 (87.7)	
Depression (GDS^c^), mean (SD)	0.27 (0.54)		0.24 (0.46)	
Loneliness (UCLA^d^), mean (SD)	0.70 (1.15)		0.56 (0.98)	
Cognitive status (TICSm^e^), mean (SD)	39.32 (3.33)		39.01 (3.03)	
**Subjective cognitive concerns**				
CFI^f^ total score, mean (SD)	1.86 (1.70)		1.96 (1.80)	
CFI-Cog^g^, mean (SD)	1.39 (1.35)		1.96 (1.80)	
Protocol adherence, mean (SD)	22.45 (2.58)		21.86 (2.30)	
**Protocol satisfaction**				
Who had fun, n (%)	102 (85)		59 (83.1)	
Who became bored, n (%)	30 (24.8)		17 (23.6)	
**Protocol motivation**				
Who were motivated, n (%)	93 (77.5)		53 (73.6)	
**M2C2^h^ task performance (average over 8-day protocol)**				
Prices, mean (SD)	0.75 (0.09)		0.75 (0.09)	
Color Shapes, mean (SD)	2.45 (0.76)		2.46 (0.80)	
Symbol Search, mean (SD)	2224.78 (447.76)		2215.08 (410.92)	

a Aβ: amyloid.

b PET: positron emission tomography.

c GDS: Geriatric Depression Scale.

d UCLA: University of California, Los Angeles.

e TICSm: modified Telephone Interview for Cognitive Status.

f CFI: Cognitive Function Instrument.

g CFI-Cog: Cognitive Function Instrument cognitive items.

h M2C2: Mobile Monitoring of Cognitive Change.

Bivariate associations are presented in Table 2. The CFI did show a small to moderate correlation with worse cognitive status (TICSm, *r*=−0.202, *P*=.03), greater loneliness (UCLA Loneliness Scale, *r*=0.248, *P*=.006), and worse adherence (*r*=−0.207, *P*=.02). In terms of overall M2C2 performances, the CFI showed a small correlation with episodic memory (ie, Prices) performance (*r*=−0.196, *P*=.03).

**Table 2.** Bivariate associations among demographic variables, subjective cognitive concerns, mobile app protocol metrics, and amyloid (Aβ) positron emission tomography (PET)^a^.

		Age	Sex	Education	Depression	Loneliness	TICSm^b^	CFI^c^	CFI-Cog^d^	Adherence	Prices	Color Shape	Symbol Search	Aβ positive
Age														
	*r*	—^e^	−0.143	−0.005	−0.154	−0.024	−0.262	0.001	−0.020	−0.060	−0.369	−0.308	0.418	0.258
	*P* value	—	.12	.95	.09	.79	.004	.99	.83	.51	<.001	<.001	<.001	.03
Sex														
	*r*	−0.143	—	−0.066	−0.038	0.146	0.184	0.001	−0.050	0.031	0.221	0.271	−0.093	0.012
	*P* value	.12	—	.47	.68	.11	.04	.99	.60	.73	.01	.003	.31	.92
Education														
	*r*	−0.005	−0.066	—	0.011	−0.085	0.218	0.074	0.083	0.036	−0.028	0.020	−0.140	−0.015
	*P value*	.95	.47	—	.91	.36	.02	.42	.37	.70	.76	.83	.13	.90
Depression														
	*r*	−0.154	−0.038	0.011	—	0.170	0.063	0.040	0.064	0.054	0.202	−0.133	−0.028	0.052
	*P value*	.09	.68	.91	—	.06	.50	.67	.49	.56	.03	.15	.76	.66
Loneliness														
	*r*	−0.024	0.146	−0.085	0.170	—	−0.056	0.248	0.104	−0.094	0.001	0.033	−0.069	−0.031
	*P value*	.79	.11	.36	.06	—	.55	.006	.26	.31	.99	.72	.45	.80
TICSm														
	*r*	−0.262	0.184	0.218	0.063	−0.056	—	−0.202	−0.183	0.008	0.350	0.225	−0.216	−0.175
	*P value*	.004	.04	.02	.50	.55	—	.03	.05	.94	<.001	.01	.02	.14
CFI														
	*r*	0.001	0.001	0.074	0.040	0.248	−0.202	—	0.941	−0.176	−0.196	−0.028	−0.023	−0.002
	*P value*	.99	.99	.42	.67	.006	.03	—	<.001	.05	.03	.76	.80	.98
CFI-Cog														
	*r*	−0.020	−0.050	0.083	0.064	0.104	−0.183	0.941	—	−0.207	−0.171	−0.017	−0.038	−0.041
	*P value*	.83	.60	.37	.49	.26	.05	<.001	—	.02	.06	.86	.68	.73
Adherence														
	*r*	−0.060	0.031	0.036	0.054	−0.094	0.008	−0.176	−0.207	—	0.221	0.043	−0.075	0.210
	*P value*	.51	.73	.70	.56	.31	.94	.05	.02	—	.02	.64	.42	.08
Prices														
	*r*	−0.369	0.221	−0.028	0.202	0.001	0.350	−0.196	−0.171	0.221	—	0.309	−0.139	−0.325
	*P value*	<.001	.01	.76	.03	.99	<.001	.03	.06	.02	—	<.001	.13	.005
Color Shapes														
	*r*	−0.308	0.271	0.020	−0.133	0.033	0.225	−0.028	−0.017	0.043	0.309	—	−0.287	−0.141
	*P value*	<.001	.003	.83	.15	.72	.01	.76	.86	.64	<.001	—	.001	.24
Symbol Search														
	*r*	0.418	−0.093	−0.140	−0.028	−0.069	−0.216	−0.023	−0.038	−0.075	−0.139	−0.287	—	0.011
	*P value*	<.001	.31	.13	.76	.45	.02	.80	.68	.42	.13	.001	—	.93
Aβ positive														
	*r*	0.258	0.012	−0.015	0.052	−0.031	−0.175	−0.002	−0.041	0.210	−0.325	−0.141	0.011	—
	*P value*	.03	.92	.90	.66	.80	.14	.98	.73	.08	.005	.24	.93	—

a The Aβ PET data was only available for a subsample (n=73).

b TICSm: modified Telephone Interview for Cognitive Status

c CFI: Cognitive Function Instrument

d CFI-Cog: Cognitive Function Instrument cognitive items

e Not applicable

### Aim 1: Subjective Cognitive Concerns and Adherence to Mobile App–Based Cognitive Assessment Protocol

Overall, remote assessment protocol adherence was high in the sample, with participants completing on average 93.5% of the 24 testing sessions over the 8-day period. CFI-Cog endorsement significantly predicted overall remote testing adherence over 8 days. This suggests that higher levels of SCC were associated with worse adherence (a diminished number of test sessions completed across the protocol) (ß=−.197, *P*=.04, 95% CI −.647 to −.021). However, this association was not observed when using the CFI total score as the predictor (Table 3).

**Table 3.** Association between subjective cognitive concerns and mobile app metrics.

	*R* ^2^	ß/aOR^a^ (95% CI)	*P* value
**Aim 1: Subjective cognitive concerns and adherence to app-based cognitive assessment protocol, ß**			
**Adherence**	0.042		
SCC^b^ (CFI^c^)		−.157 (−.459 to .042)	.10
Age		−.046 (−.106 to .064)	.63
Sex		.051 (−.657 to 1.150)	.59
Depression (GDS^d^)		.072 (−.479 to 1.068)	.45
Loneliness (UCLA^e^)		−.071 (−.5.20 to .239)	.47
**Aim 2: Subjective cognitive concerns, satisfaction, and motivation on app-based cognitive assessment, aOR**			
**Protocol satisfaction**			
**Level of fun**	0.060		
SCC (CFI)		1.016 (0.738 to 1.398)	.92
Age		0.938 (0.844 to 1.042)	.23
Sex		0.328 (0.112 to 0.963)	.04
Depression (GDS)		0.679 (0.28 to 1.606)	.38
Loneliness (UCLA)		0.883 (0.554 to 1.408)	.60
**Boredom**	0.026		
SCC (CFI)		1.013 (0.783 to 1.310)	.92
Age		1.056 (0.966 to 1.155)	.23
Sex		0.575 (0.213 to 1.552)	.27
Depression (GDS)		1.060 (0.473 to 2.375)	.89
Loneliness (UCLA)		1.130 (0.785 to 1.625)	.12
**Protocol motivation**			
**Motivation**	0.028		
SCC (CFI)		0.891 (0.691 to 1.148)	.37
Age		0.962 (0.877 to 1.054)	.41
Sex		1.689 (0.613 to 4.603)	.31
Depression (GDS)		0.685 (0.312 to 1.503)	.35
Loneliness (UCLA)		1.286 (0.831 to 1.991)	.26
**Aim 3: Subjective cognition and performance on app based cognitive assessment, ß**			
**Prices**	0.234		
SCC (CFI)		−.200 (−.020 to −.002)	.02
Age		−.318 (−.009 to −.003)	<.001
Sex		.193 (.005 to .070)	.02
Depression (GDS)		.174 (.001 to .056)	.04
Loneliness (UCLA)		−.013 (−.015 to .013)	.88
**Color Shapes**	0.170		
SCC (CFI)		−.016 (−.085 to .071)	.86
Age		−.301 (−.073 to −.020)	<.001
Sex		.205 (.051 to .612)	.02
Depression (GDS)		−.181 (−.489 to −.009)	.04
Loneliness (UCLA)		.030 (−.098 to .138)	.74
**Symbol Search**	0.178		
SCC (CFI)		−.007 (−48.34 to 44.72)	.94
Age		.416 (22.302 to 53.977)	<.001
Sex		−.027 (−192.599 to 140.950)	.76
Depression (GDS)		.041 (−109.610 to 176.575)	.64
Loneliness (UCLA)		−.045 −92.465 to 55.043	.62

a aOR: adjusted odds ratio.

b SCC: subjective cognitive concern.

c CFI: Cognitive Function Instrument.

d GDS: Geriatric Depression Scale.

e UCLA: University of California, Los Angeles.

### Aim 2: Subjective Cognitive Concerns, Satisfaction, and Motivation on Mobile App–Based Cognitive Assessment

In terms of overall protocol satisfaction, participants endorsed that completing the assessments was fun (n=102, 85%) and not boring (n=92, 75.2%). Participants also reported that they did not encounter difficulties in motivating themselves to give their best performance (n=93, 77.5%). CFI total score was not associated with metrics of study satisfaction in the adjusted models, including how fun or boring participants found the protocol to be. In terms of motivation, CFI total score was not predictive of self-reported effort across the protocol period (more details in Table 3).

### Aim 3: Subjective Cognitive Concerns and Performance on Mobile App–Based Cognitive Assessment

SCCs and their association with overall objective cognitive performances on digital cognitive tests was also investigated (more details in Table 3). High SCC endorsement on the CFI total score was significantly predictive of poorer overall performance on the Prices task (ß=−.200, *P*=.02, 95% CI −.020, to −.002). There were no significant associations between the CFI and performance metrics for Color Shapes (*P*=.86) or Symbol Search (*P*=.94).

**Figure 3.**
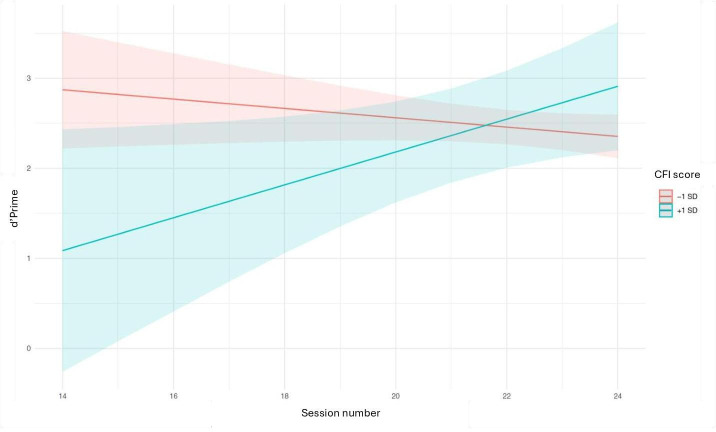
Subjective cognitive concerns association with working memory longitudinally over the 8-day mobile-appmobile app protocoll. CFI: Cognitive Function Instrument.

We also investigated the association of SCC and longitudinal trends of cognitive performance over the 8-day testing period (M2C2 app performance descriptives for each study day have been previously reported) [38]. There was a main effect of the CFI total score suggesting that higher levels of SCC were associated with worse performance on Color Shapes (working memory) at day 1 (unstandardized estimate [b]=−1.047, SE=0.47, *P*=.03). Furthermore, there was a significant interaction between SCCs and Color Shapes performance (b=0.048, SE=0.02, *P*=.03), such that higher CFI total scores were associated with improved performance on this subtest over time. In other words, although high SCC was associated with worse working memory performance initially, individuals seemed to benefit from repeated practice and ultimately performed better (more details in Figure 3). There were no significant associations between SCC and intraindividual variability in Prices or Symbol Match performances over the protocol period.

### Secondary Aim: Moderating Effect of Amyloid Status on the Relationship Between Subjective Cognitive Concerns and Mobile App–Based Cognitive Assessment

The moderation of Aβ positivity on the relationship between CFI and app-based metrics, as well as CFI and cognitive performance (ie, Prices, Color Shapes, and Symbol Search) was investigated in a subset of individuals with Aβ PET scans (n=73). The presence of Aβ did not moderate the relationship between CFI total score and adherence. There was evidence for moderation at the *P*<.10 level of Aβ positivity on the relationship between CFI total score and Prices was observed, but did not fall within the α=.05 threshold (ß=.179, *P*=.09, 95% CI −.003 to .036). Aβ status did not moderate the association between SCC and Color Shapes or Symbol Search.

## Discussion

### Principal Findings

This study sought to characterize the relationship between SCC and mobile app–based protocol engagement (adherence, satisfaction, and motivation) and performance on cognitive tasks in a cohort of cognitively healthy older adults. While protocol satisfaction and motivation were not impacted by SCCs, overall adherence was associated, such that higher levels of SCCs predicted lower rates of adherence. Overall, we show that SCCs were associated with worse objective performance on mobile app–based assessments. Our findings suggest that mobile app–based assessments hold promise as sensitive tools to detect subtle neurodegenerative changes in individuals at risk for dementia, with potential application for early detection, diagnosis, and treatment.

We observed an association between SCCs, as measured on the Cognitive Function Index (CFI), and overall worse performance on a test of episodic memory (Prices). A significant association was not observed between SCC and overall performance on tasks of processing speed or working memory. Findings in the broader SCD literature are quite mixed as to whether SCCs are or are not associated with concurrent cognitive functioning abilities [52]; as such, our results may indicate that the use of sensitive digital cognitive tools could enhance the association between SCC and objective cognitive metrics. Our result reflecting a unique association with episodic memory is perhaps clinically meaningful, as memory concerns specifically are frequently considered to be the hallmark presenting symptom of AD [2,53], particularly in individuals over the age of 65 [53]. This observation is underscored by our finding of a moderating effect of cerebral Aβ positivity (at the *P*<.10 level) on the relationship between SCC and episodic memory (Prices) in a subsample of participants with biomarkers available for analysis (secondary aim), such that the association of SCC with episodic memory was stronger in the presence of Aβ. This result adds to the growing evidence base for an association between SCC and higher Aβ [52]. Replication of this analysis in a larger sample may clarify the interplay between SCC, AD biomarkers, and subtle memory deficits observed on mobile app–based assessment in otherwise cognitively and clinically normal older adults. That being said, it is worth considering that the unique association between SCC and episodic memory in our sample may be at least somewhat secondary to the way the CFI is constructed, as this instrument mostly focuses on episodic memory-related cognitive concerns [45]. Previous studies have demonstrated the importance of querying beyond just memory concerns to most sensitively detect dementia risk [54,55]. Indeed, SCC can be sampled by a multitude of techniques and approaches (eg, memory only vs multiple cognitive domains; traditional paper and pencil vs digital assessments; self vs informant report; capturing current ability vs change in ability, etc) [24,25], all of which may impact the sensitivity of the SCC measures to detect current and risk for future cognitive impairment. Future research should carefully weigh SCC assessment approaches, and consider incorporating a more comprehensive SCC screener which queries across a broader range of cognitive domains, as this may be valuable for detecting more meaningful associations between SCC and mobile app–based cognitive performances.

We also evaluated patterns of cognitive performance across the 8-day assessment period. Analyses revealed that more SCC was associated with initially worse working memory (Color Shapes) performance on day one. However, individuals with higher SCC showed progressively improving working memory performance over the assessment period, and by day 8 were performing in a similar range with those with lower SCC. This pattern was not observed on the episodic memory or processing speed subtests. This finding is consistent with recent results from Aschenbrenner et al [56], which showed variability over high frequency app-based assessments in attention and working memory-based skills, but not in episodic memory, in older adults who were at increased genetic risk for AD [56]. Our finding of an initial relative weakness in working memory skills could reflect the presence of mild cognitive inefficiencies in older adults with SCC, which may be compensated for with repeated exposure to the task over time. This may be due to early dysfunction in working memory processes, which have been characterized in preclinical AD [57,58]. Subjectively, this may be experienced by these individuals as thinking which requires increased concentration, that is punctuated by occasional lapses, and is associated perhaps with a sense of being cognitively overwhelmed, all of which may be driving the report SCC in daily life, but may be subtle enough that it may not be detected on traditional neuropsychological tests. Hence, this type of unique marker of cognitive inefficiency would only be afforded through high frequency, mobile app–based cognitive assessment in this population. The plausible role of performance anxiety may also explain variability in cognitive performance overtime. Although beyond the scope of the present analysis, research which comprehensively measures the role of in-the-moment psychological and physiological states by ecological momentary assessment techniques in relation to in the moment SCC and objective cognition will be critical next steps.

Our findings related to SCC and metrics of protocol engagement and satisfaction with unsupervised, high frequency digital cognitive assessments have potentially broad implications for aging research, especially in light of increasing interest in using these techniques to remotely assess cognitive functioning in this population [31,32]. Indeed, whether or not a study is directly examining SCC or includes SCD as a clinical group of interest, aging studies must contend with the fact that SCCs are widespread and may be clinically meaningful feature among the older adults in their sample [2]. We show that SCC was not associated with protocol satisfaction (including how fun or how boring the experience was) nor did SCC effect how motivated a participant was to engage and put forth their best effort. However, higher levels of SCC did impact protocol adherence in our sample, with mildly worse compliance those with higher SCC observed. For context, protocol adherence was overall quite high in our population (93.5%) and similarly high compliance rates have been described in several other recent smartphone-based studies [32], yet no previous studies have directly explored the impact of SCCs on protocol adherence. It may be that high frequency, mobile assessments are able to detect mild patterns of forgetfulness (manifested in slightly worse adherence rates) in older adults with SCC, so future research should be attuned to this possibility. Replication of this association between SCC and adherence to remote protocols in larger, more demographically diverse samples is warranted.

### Limitations

Some limitations of this study, as well as potential future directions of this research, warrant acknowledgment. Our sample was largely White, disproportionally female, and highly educated. This limits the generalizability of the study findings to broader demographic groups. Our sample was from a relatively small single cohort and only a subset had AD biomarkers available for analysis, restricting study power and may have impeded our ability to obtain significant findings. A measure of anxiety symptomology was not available in the present sample, limiting our ability to understand the plausible role of anxiety, SCC, and performance on digital assessments [32,59]. The main predictor of interest in this study, the CFI, shows adequate evidence for validity and reliability [42], but is somewhat limited in the domains of cognitive concerns it measures (eg, memory and daily functioning). Research has shown that comprehensive assessment of concerns across a broad range of cognitive domains may be most sensitive to risk in aging populations [60], so future smartphone-based research may consider including a broader range of cognitive concern items to better understand this relationship. We were unable to assess informant-report of SCC in this sample, which has been demonstrated to be optimized to predict risk above and beyond self-report [25], and this should be addressed in future smartphone studies. Although such metrics were not available in this study, there is also a small but growing literature which has examined the use of smartphones to assess SCC in a digital remote format in tandem with cognitive tests and relevant clinical outcomes in older adult samples [61-63]. Future research on SCC and smartphone-based digital cognitive assessments should investigate the relative value of traditional in person reported SCC (eg, the CFI) versus smartphone based digital SCC assessments. Finally, this cross-sectional study was not able to investigate the relationship between SCC and longitudinal decline on smartphone-based cognitive performance in individuals with positive AD biomarkers. It will be essential for future studies to follow participants over time to understand if SCC at baseline may predict incident decline on these novel cognitive metrics. Due to the limited nature of this pilot study, we cannot provide specific recommendations about optimal SCC assessment approach for use in future digital cognitive studies.

### Conclusions

SCCs are frequently one of the earliest clinical symptoms of dementia [2]. However, clinical interpretations of SCCs are complicated by the ubiquitous endorsement of these concerns [2], their potential for non-neurodegenerative etiology (eg, psychiatric, medical, or sociodemographic factors) [2,3], and frequent lack of association with traditional objective cognitive testing [1]. Smartphone-based, digital cognitive assessments, which are increasingly used in aging research, may offer potential for improved ecological validity and sensitivity to subtle markers of cognitive decline [30-32,35]. Hence, such smartphone-based digital tools may be able to capture quite mild deficits in individuals with SCC who perform within normal limits on traditional neuropsychological tests. Results from the current study showed that SCCs are associated with worse overall memory performance on mobile app assessment, and patterns of cognitive inefficiency (variable working memory) and mild forgetfulness (diminished adherence) across an 8-day assessment period in cognitively intact older adults. Findings indicate that mobile app assessments may be uniquely sensitive to very subtle neurodegenerative changes in at risk older adults, with critical implications for early detection and timely intervention.

## Abbreviations

AD: Alzheimer disease
ADRD: Alzheimer disease and related dementias
Aβ: amyloid
CFI: Cognitive Function Instrument
CFI-Cog: Cognitive Function Instrument cognitive items
GDS: Geriatric Depression Scale
M2C2: Mobile Monitoring of Cognitive Change
MAP: Memory and Aging Program
PET: positron emission tomography
SCC: subjective cognitive concern
SCD: subjective cognitive decline
TICSm: modified Telephone Interview for Cognitive Status
UCLA: University of California, Los Angeles
